# Supplementary figures and images for: Membrane potential modulates ERK activity and cell proliferation in human cells
Source: eLife. 2025 Nov 14;13:RP101613. doi: 10.7554/eLife.101613 (PMC12618006; doi:10.7554/eLife.101613)

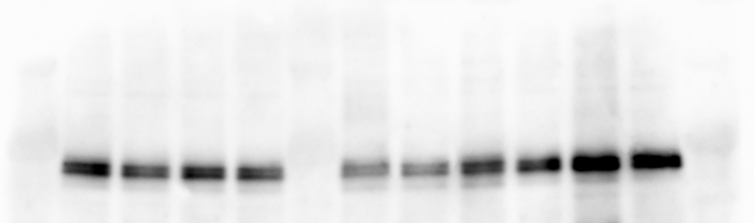

Supplement: Figure 4—source data 1. [file elife-101613-fig4-data1.zip › Figure4_source data 1/phspho cRaf.tif]

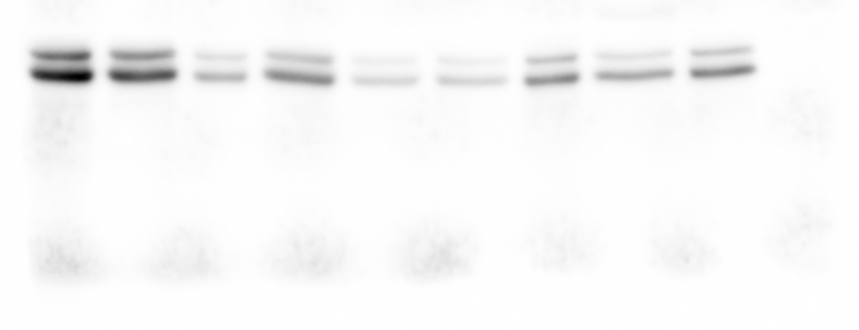

Supplement: Figure 4—source data 1. [file elife-101613-fig4-data1.zip › Figure4_source data 1/phspho ERK.tif]

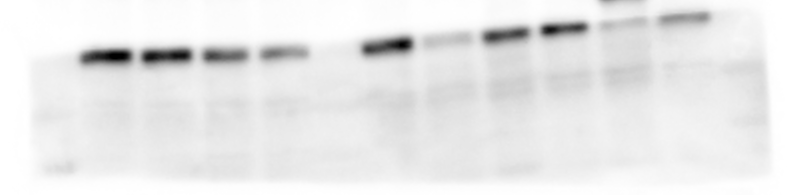

Supplement: Figure 4—source data 1. [file elife-101613-fig4-data1.zip › Figure4_source data 1/phspho MEK.tif]

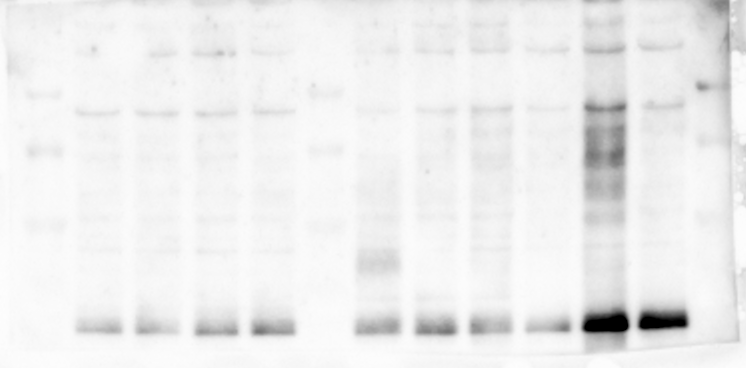

Supplement: Figure 4—source data 1. [file elife-101613-fig4-data1.zip › Figure4_source data 1/total cRaf.tif]

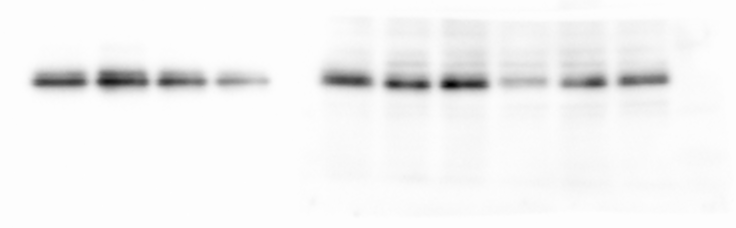

Supplement: Figure 4—source data 1. [file elife-101613-fig4-data1.zip › Figure4_source data 1/total ERK.TIF]

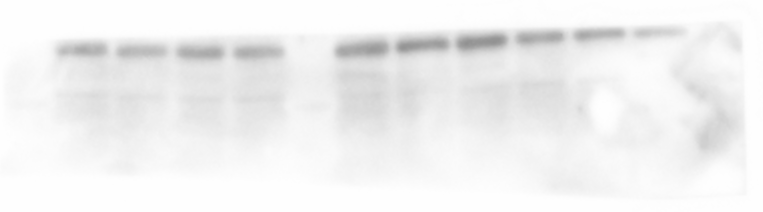

Supplement: Figure 4—source data 1. [file elife-101613-fig4-data1.zip › Figure4_source data 1/TotalMEK.tif]

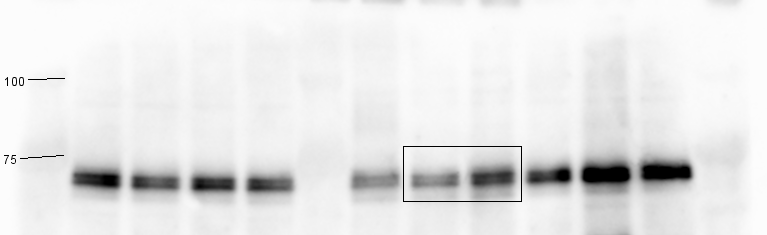

Supplement: Figure 4—source data 2. [file elife-101613-fig4-data2.zip › Figure4_source data 2/phospho cRaf labelled.tif]

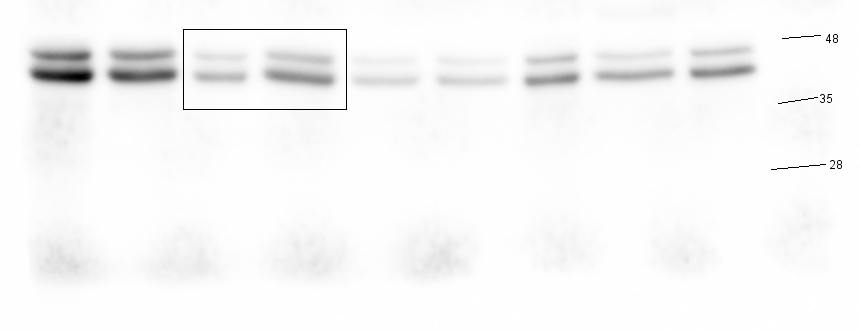

Supplement: Figure 4—source data 2. [file elife-101613-fig4-data2.zip › Figure4_source data 2/phospho ERK labelled.tif]

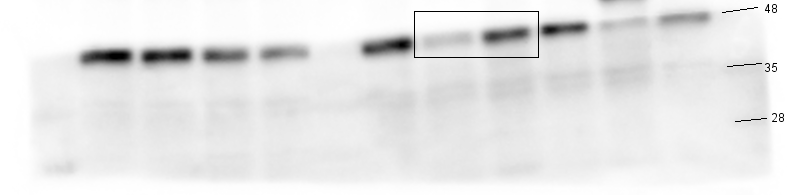

Supplement: Figure 4—source data 2. [file elife-101613-fig4-data2.zip › Figure4_source data 2/phospho MEK labelled.tif]

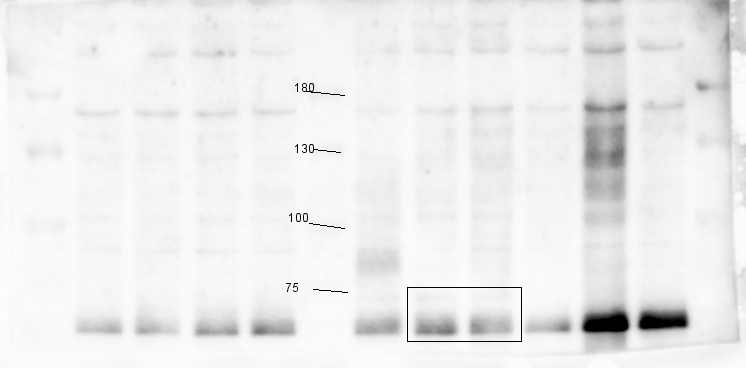

Supplement: Figure 4—source data 2. [file elife-101613-fig4-data2.zip › Figure4_source data 2/total cRaf labelled.tif]

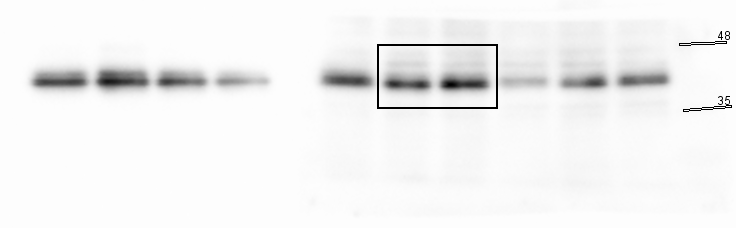

Supplement: Figure 4—source data 2. [file elife-101613-fig4-data2.zip › Figure4_source data 2/total ERK labelled.tif]

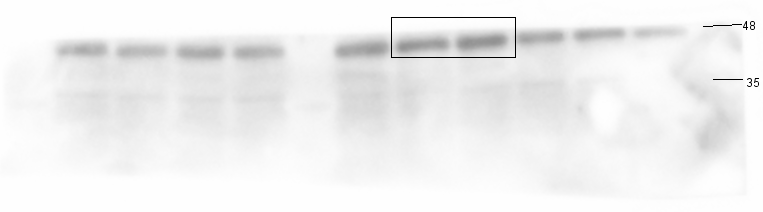

Supplement: Figure 4—source data 2. [file elife-101613-fig4-data2.zip › Figure4_source data 2/TotalMEK labelled.tif]
