# Supplementary material for: Membrane potential modulates ERK activity and cell proliferation in human cells
Source: eLife. 2025 Nov 14;13:RP101613. doi: 10.7554/eLife.101613 (PMC12618006; doi:10.7554/eLife.101613)
Supplement: MDAR checklist [file elife-101613-mdarchecklist1.pdf]

MDAR Checklist (Materials Design Analysis Reporting)

Manuscript Title: Membrane potential modulates ERK activity and cell proliferation in human cell

Corresponding Author: Mari Sasaki

---

1. Materials

Reagent type (species) or resource	Designation	Source or reference	Identifiers	Additional information
Cell line (Homo sapiens)	U2OS	European Collection of Authenticated Cell Cultures (ECACC)	Cat# 92022711	Authenticated by STR profiling; mycoplasma-negative
Cell line (Homo sapiens)	HEK293T	RIKEN Cell Bank (Tsukuba, Japan)	RCB2202	Authenticated by STR profiling; mycoplasma-negative
Cell line (Homo sapiens)	A431	RIKEN Cell Bank (Tsukuba, Japan)	RCB0202	Authenticated by STR profiling; mycoplasma-negative
Cell line (Homo sapiens)	HeLa	RIKEN Cell Bank (Tsukuba, Japan)	RCB0007	Authenticated by STR profiling; mycoplasma-negative
Plasmid	EKAREV	Gift from Dr. Michiyuki Matsuda (Kyoto University)	—	FRET-based ERK activity reporter

Plasmid	Raichu-Ras	Gift from Dr. Michiyuki Matsuda (Kyoto University)	—	FRET-based Ras activity reporter
Plasmid	mRFP-Lact-C2	Addgene	Plasmid #74061; RRID:Addgene_74061	Gift from Sergio Grinstein
Plasmid	YFP-Lact-C2	This study	—	Generated by replacing mRFP with YPet
Plasmid	CFP-Lact-C2	This study	—	Generated by replacing mRFP with SECFP
Chemical compound	U0126	Promega	V112A	MEK inhibitor
Chemical compound	Recombinant human EGF	Thermo Fisher Scientific	Cat# PHG0311	Growth factor stimulation
Chemical compound	Fendiline	Cayman Chemical	Cat# 17295	Lipid metabolism modulator
Chemical compound	Gramicidin	Sigma-Aldrich	Cat# G5002	Ionophore for depolarization
Antibody	anti-ERK1/2 (total ERK)	Santa Cruz Biotechnology	Cat# SC-514302	WB (1:1000)
Antibody	anti−phospho-ERK (Thr202/Tyr204)	Cell Signaling Technology	Cat# 4370	WB (1:1000)
Antibody	anti-MEK1/2 (total)	Cell Signaling Technology	Cat# 4394	WB (1:1000)
Antibody	anti−phospho-MEK (Ser217/221)	Cell Signaling Technology	Cat# 2338	WB (1:1000)
Antibody	anti−c-Raf (total)	Cell Signaling Technology	Cat# 9422	WB (1:1000)
Antibody	anti−phospho−c-Raf (Ser338)	Cell Signaling Technology	Cat# 9431	WB (1:1000)

2. Design

- Sample sizes, replicates, and experimental conditions are described in Materials & Methods or Figure legend.
- Randomization and blinding were not applicable for live-cell imaging and Western blot experiments.
- All experiments were performed under identical culture and treatment conditions to allow direct comparison.

---

3. Analysis

- Image quantification was performed using ImageJ.
- Statistical analyses are described in figure legends.
- Raw Western blots are provided as source data files.

---

4. Reporting

All data generated or analyzed are included in the article and source data files.

- MDAR checklist is provided as part of the submission.
- Reagent sourcing details are included in Materials & Methods.
